# Oral colon-targeted delivery of recombinant human MANF for alleviation of ulcerative colitis

**DOI:** 10.1016/j.ijpx.2025.100320

**Published:** 2025-02-26

**Authors:** Jie Zhou, Tian-Le Li, Bo Wei, Yue-Feng Ruan, Ye-Qin Wang, Jiao-Yan Liu, Meng-Meng Song, Yu-Xian Shen

**Affiliations:** a School of Basic Medical Sciences, Anhui Medical University, 81 Meishan Road, 230032 Hefei, Anhui, PR China; b Anhui Provincial Institute of Translational Medicine, 230032 Hefei, Anhui, PR China

**Keywords:** MANF, Oral delivery, Colon targeted, Hydrogel, Ulcerative colitis

## Abstract

Midbrain astrocyte-derived neurotrophic factor (MANF) is a secreted protein induced by endoplasmic reticulum stress. Previous studies have indicated that intravenous administration of 1 mg/kg/day recombinant human MANF protein with His tag (His-MANF) for 3 days can ameliorate acute ulcerative colitis in mice. However, long-term intravenous therapy has many disadvantages. In this paper, His-MANF protein was successfully encapsulated into alginate and hyaluronic acid hybrid hydrogel microcapsules in one step using the gas shear method and then coated by Eudragit S100 to construct an oral colon-targeted delivery system (MSH@E). The MSH@E microcapsules exhibited controlled and sustained release behavior and colon-targeting properties. Both fluorescent imaging and immunohistochemistry staining results showed that His-MANF protein could accumulate in the colitis colon for a longer residence time after oral delivery. In vivo studies demonstrated that oral administration of MSH@E microcapsules could alleviate DSS-induced colitis in mice without systemic toxicity. Importantly, even if the oral His-MANF dose was half of the intravenous His-MANF dose, oral delivery was still much more effective than intravenous injection, suggesting the development of the oral colon-targeted delivery system (MSH@E) has great significance and makes a breakthrough from intravenous to oral administration for His-MANF treatment of ulcerative colitis (UC).

## Graphical abstract

**Figure:**
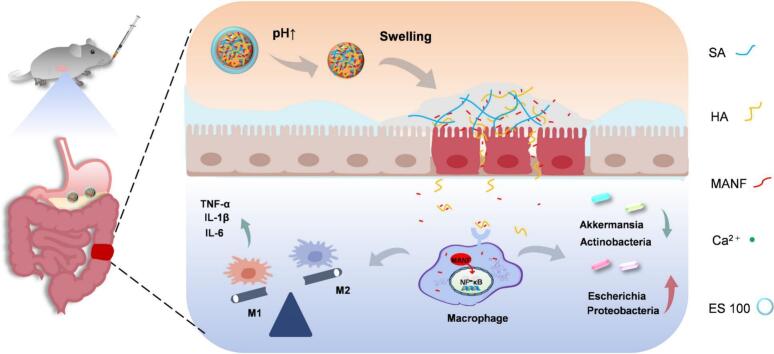

## 1 Introduction

Ulcerative colitis (UC) is a recurring, chronic inflammatory illness of the colon and rectum, which is a primary subtype of intestinal bowel disease (IBD) (Kaplan, 2015; Nakase et al., 2021; Ungaro et al., 2017). IBD has been demonstrated to be related to many factors, such as immune system dysfunction, genetic predisposition, dysregulation of floras, pathogenic infection, and environmental factors (Ng et al., 2017; Ramos and Papadakis, 2019). Treatment of IBD aims to control symptoms (Liu et al., 2023). Many small-molecular drugs (e.g. corticosteroids, aminosalicylates and immunomodulators) are available for IBD patients in clinics (Bressler et al., 2015; Kotla and Rochev, 2023). Nevertheless, systemic administration of these drugs usually has a risk of severe side effects and fails to prevent recurrence (Sandborn et al., 2018; Torres et al., 2016).

In the last several decades, many biologic therapeutics (peptide- and protein-based medicines) have become the choice when conventional therapies fail, which brought a new sight of treatment of IBD. Infliximab (IFX) was the first biologic approved for UC in 2005 (Lichtenstein et al., 2006). Since then, numerous biologics, including adalimumab, golimumab, vedolizumab, and ustekinumab had been authorized (Liu et al., 2023). Although conventional therapies initially respond to most patients, IBD cannot be cured, and patients still need biologics to further control the disease in the long term (Baumgart et al., 2019). Compared with traditional drugs, biologics have greatly reduced the serious side effects that are caused by long-term use and improved the quality of life of patients (Liu et al., 2023). More importantly, due to the limited remission rate and persistence of some classical biologics, the development of other biologics is also highly competitive in the future (AlAmeel et al., 2023).

Mesencephalic astrocyte-derived neurotrophic factor (MANF) is a secreted protein induced by endoplasmic reticulum (ER) stress (Apostolou et al., 2008). It has been reported that Parkinson's disease, Alzheimer's disease, stroke, retinal regeneration, diabetes, and other diseases linked to ER stress may benefit from treatment of MANF (Danilova et al., 2019; Hakonen et al., 2018; Neves et al., 2016; Xu et al., 2019; Yu et al., 2021). Recently, our group found that MANF-specific knockout could up-regulate the expression of BATF2, promote the recruitment of Ly6C^hi^CX3CR1^int^ inflammatory macrophages and the aggregation of Th17 cells, which led to the apoptosis of intestinal epithelial cells and further aggravated colon inflammation (Yang et al., 2023). It was also demonstrated that intravenous administration of recombinant human His-MANF protein significantly alleviated acute ulcerative colitis in mice by regulating the activation of congenital immune inflammatory response (Yang et al., 2023). However, all the preclinical studies of the application of MANF are based on local injection and intravenous injection (Hou et al., 2023; Zeng et al., 2023; Zhang et al., 2022).

Oral administration is the preferred method of administration for patients with chronic gastrointestinal disease for its convenience, safety, and rapid access to the colon (Sung et al., 2022). However, the pH of the gastrointestinal tract (GIT) varies greatly: the stomach (1 to 2.5), the small intestine (5.5 to 7), and the large intestine (5.5 to 7.5) (Durán-Lobato et al., 2020; Evans et al., 1988). In addition, there are also a lot of enzymes metabolized by various species of microorganisms (Gamboa and Leong, 2013). In such a harsh gastrointestinal environment, it is challengeable to maintain normal function of drugs, especially for protein drugs. Therefore, it is of great significance to construct an oral colon-targeted delivery system to accelerate its clinical application.

Oral delivery carriers including lipid nanoparticles (Fukuta et al., 2019), mesoporous silicon (Liu et al., 2022), and hydrogels (Ouyang et al., 2023a; Wu et al., 2023) have been investigated for many years. Hydrogels have several advantages including high swelling and excellent biocompatibility (Zhu et al., 2022). Biomolecules are encapsulated through the hydrogel's three-dimensional network structure without chemical bonding with the loaded molecules. The network structure protects the drug from enzymatic degradation and allows for effective release, thus preserving the activity of drug molecules. Sodium alginate (SA) is a biodegradable natural anionic polymer with bio-adhesive properties (Pi et al., 2022). SA hydrogel can be quickly formed when cations such as Ca^2+^ present, Na^+^ on G unit will undergo an ion exchange reaction with bivalent cations, and the G unit will accumulate to form a cross-linked network structure. The mild condition avoids the inactivation of active substances such as sensitive drugs and proteins. Additionally, SA has obvious pH sensitivity. The ionization degree of the carboxy group decreases and the molecular chain shrinks under acidic conditions, while the carboxy group is continuously dissociated, and the molecular chain is extended under alkaline conditions. Therefore, it can be stable in the stomach, swell in the colon, and be metabolized by colonic microorganisms, which is often used for colon oral delivery. (Agüero et al., 2017; Ouyang et al., 2023a).

Hyaluronic acid (HA) is a natural polysaccharide and has good gelling properties (Bayer, 2020). The interaction between HA and the membrane protein CD44 has been well studied, leading to a great deal of research in targeted drug delivery (Lee et al., 2020; Xu et al., 2022; Zhang et al., 2023). It has also been found that HA can suppress inflammatory responses, promote the repair of damaged mucosa, and decrease intestinal permeability (Lee et al., 2020; Ouyang et al., 2023b). Therefore, we intended to introduce HA into SA to obtain SA/HA hybrid hydrogel microcapsules. This hybrid hydrogel is expected to enhance its affinity to the inflamed colon via binding with the CD44 receptor of inflammatory epithelial cells and inflammatory macrophages (Zhang et al., 2023).

To further increase stability in the gastric acid environment, polymers based on poly-methacrylate are usually used to coat the drug carrier. Eudragit S 100, a pH-sensitive polymer that dissolves in the more alkaline environment of the terminal ileum, which is a kind of common enteric-coated material. Therefore, encapsulating drug-loaded SH/HA hybrid hydrogel microcapsules with Eudragit S 100 can further enhance their stability in the upper gastrointestinal tract (GIT).

**Scheme. 1:**
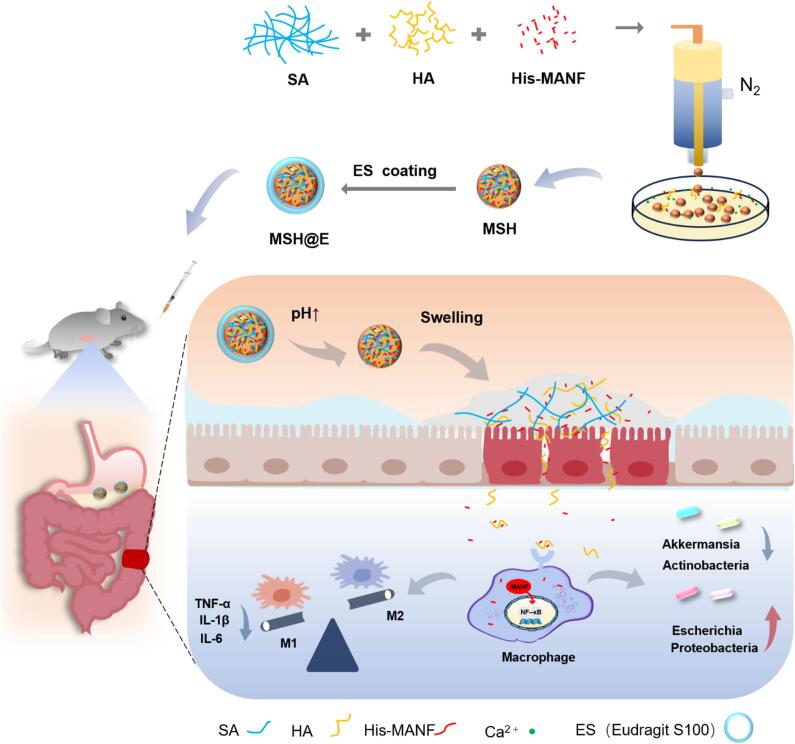
The schematic diagram of the preparation and possible mechanism of MSH@E microcapsules for alleviating UC. MSH@E microspheres were prepared following two steps: First, the mixture of SA, HA, and His-MANF protein was added to a gas flow shearing device. MSH microspheres with different particle sizes can be obtained by adjusting the gas flow rate. Then, MSH hydrogel microspheres were coated with a Eudragit S100 layer to obtain MSH@E microcapsules. After oral administration, MSH@E microcapsules kept stable in the stomach and swelled in the intestine. With the aid of HA-CD44 interaction, the swelled hydrogel mainly adhered to the inflamed area. Then His-MANF protein promoted macrophage polarization towards anti-inflammatory M2 phenotypes, reduced the secretion of inflammatory factors, alleviated intestinal damage, and improved intestinal flora dysbiosis.

In this study, His-MANF oral colon-targeted delivery system was developed. Firstly, His-MANF was encapsulated in SA/HA hybrid hydrogel microcapsules (MSH) by using one step gas-shearing method (Tang et al., 2019). Then MSH was coated by polymer Eudragit S100 (Scheme 1). Then, both in vitro and in vivo biocompatibility were evaluated. Immunohistochemistry results showed His-MANF was accumulated in the colon by oral delivery. The in vivo pharmacodynamics results showed that MSH@E microcapsules could significantly alleviate colitis in a mouse model.

## 2 Materials and methods

### 2.1 Materials

Hyaluronic acid (HA, 400–800 kDa), sodium alginate (SA, 180–220 mpa.s) and Coomassie brilliant blue were purchased from Aladdin (Shanghai, China). Bovine serum albumin (BSA, 66 kDa) was purchased from Solarbio. Lipopolysaccharide (LPS) was purchased from Sigma. Dextran sodium sulfate (DSS, 36000–50,000 Da) was purchased from MP Biomedicals (CA, USA). Sulfo-Cyanine5 NHS ester (Cy 5, R-H-5029) was purchased from Xian Ruixi Biological Technology (Xian, China). Eudragit S100 (ES100) is presented by Evonik (Darmstadt, Germany). Methylthiazolyldiphenyl-tetrazolium bromide (MTT) was purchased from Beyotime (Shanghai, China). Enteric-coated capsules were purchased from Yuyan Instruments (YG00–2, Shanghai, China). All other chemical reagents were purchased from Sinopharm Group (Shanghai, China).

### 2.2 Preparation of MSH@E microcapsules

His-MANF protein was expressed and purified as the previous reference (Yu et al., 2010). SA, HA and His-MANF protein (or BSA) with different ratios (see Table S1) were mixed and centrifuged to eliminate bubbles. Then the mixed solution was added to the gas flow shear equipment and dropped into CaCl_2_ solution under agitation and then crosslinked for 10 min. After that, the prepared His-MANF/SA/HA hydrogel microcapsules (also abbreviated as MSH) were washed with deionized water two times and then collected for quick freeze-drying. SA, SA/HA, and BSA/SA/HA hydrogel microcapsules were prepared similarly.

For Eudragit S100 coating, MSH or SH microcapsules (50 mg) and ES 100 (10 mL, 5 % *w*/*v*) were first dispersed in acetone/ethanol (10 mL, volume ratio: 2:1). Then this mixture was transferred to light liquid paraffin containing 1 % w/v span 80. After stirring at 1000 rpm for 3 h, the solvent was evaporated. Subsequently, ES 100-coated MSH microcapsules or SH microcapsules (MSH@E, SH@E) were collected by centrifugation and washed with hexane (Paharia et al., 2007). Finally, the MSH@E and SH@E microcapsules were freeze-dried and stored at −20 °C.

### 2.3 Characterization

The morphology of SA hydrogel microspheres, SA/HA hybrid hydrogel microspheres and MSH@E microcapsules were observed by optical microscope (Olympus, IX71) and scanning electron microscope (Zeiss, Sigma 300). The size of MSH@E microcapsules was analyzed by Image J software according to the optical microscope images. The rheological viscosities of the mixture of HA and SA were evaluated on a rheometer AR1000 (TA Instruments Inc.) with a 40 mm plate geometry. For determining loading efficiency (LE) and encapsulation efficiency (EE), MSH@E microcapsules were incubated in PBS for 24 h at 37 ± 0.5 °C. The protein content was determined by using the Coomassie brilliant blue method. Then LE and EE were calculated as follows:

$$\mathrm{LE}\;\left(\%\right)=W_{\text{MANF in the microcapsules}}/W_{\text{MANF and polymers added}}\times 100\%$$

$$\mathrm{EE}\;\left(\%\right)=W_{\text{MANF in the microcapsules}}/W_{\text{MANF added}}\times 100\%$$

### 2.4 In vitro drug release

For His-MANF release experiment, 100 mg MSH@E microcapsules, MSH microcapsules or MS microcapsules were exposed to simulated gastric fluid (SGF) for 2 h, simulated human intestinal fluid (SIF) for 3 h and simulated colonic fluid (SCF) for 12 h at 37 ± 0.5 °C. At the designed time point, 0.1 mL supernatant was removed to observe the morphology under the microscope and measure the concentration of His-MANF with the Bradford method. His-MANF enteric-coated capsule (MECC) was used as the control group. Finally, the released His-MANF was analyzed by SDS-PAGE.

### 2.5 Cells and animals

Mouse leukemia cells of monocyte macrophage (RAW 264.7, ATCC, USA) were cultured in RPMI 1640 medium (Gibco, USA) containing 10 % fetal bovine serum (FBS, Gibco, USA). Human colorectal cancer cell (CaCO_2_) and human normal colonic epithelial cells (NCM460, ATCC, USA) were cultured in DMEM medium (Gibco, USA) containing 10 % fetal bovine serum (FBS, Gibco, USA). Female C57BL/6 J mice aged 8–10 weeks were purchased from GemPharmatech (license number: SCXK Su 2023–0009, Nanjing, CN) and housed in cages at a temperature of 25 °C and relative humidity of approximately 40 %. All animal work was approved by the Animal Ethics Committee of Anhui Medical University.

### 2.6 Toxicity evaluation

RAW 264.7, NCM460, and CaCO_2_ cells were selected as cell models to investigate the in vitro toxicity of the drug delivery system MSH@E. In vitro toxicity evaluation was performed by using Calcein AM and propidium iodide (PI) staining kit (Beyotime, C2015S), MTT assay, and hemolysis assays (details were shown in the supporting information 1–3).

To assess systemic toxicity, 8–10 weeks female C57BL/6 J mice were orally treated with 750 mg/kg MSH@E (equivalent to 50 mg/kg of His-MANF) for 6 consecutive days. The body weight of mice was measured daily. On the 7th day, the mice were killed and the major organs (heart, liver, spleen, lung, and kidney) were collected for hematoxylin and eosin (HE) staining.

### 2.7 DSS-induced colitis model

Female C57BL/6 mice were given 3.5 % DSS in drinking water for 7 days after acclimatizing for 1 week.

### 2.8 Bio-distribution and targeting evaluation

His-MANF protein was firstly fluorescently labelled with Cy 5 and then used to prepare MS@E and MSH@E microcapsules to carry out the biodistribution study. Cy 5 labelled His-MANF was prepared as follows: 100 μL 10 mg/mL Cy5-NHS ester solution was added to 2 mg/mL His-MANF solution and then kept stirred in the dark for 30 min. Finally, the mixture was dialyzed to remove the unreacted Cy5-NHS ester and other by-products.

Healthy mice and colitis mice were grouped and orally treated with PBS, Cy 5, and MS@E or MSH@E labelled with Cy 5. Then all the mice were imaged using an in vivo imaging system (Amix, Spectral Instruments) at 2 h, 6 h, 12 h, and 24 h, respectively, and the average fluorescence intensity was also quantified. After 24 h, the mice were killed, and the major organs were collected for fluorescent imaging. Colon tissues were further sectioned to carry immunohistochemical staining using his antibody (Proteintech, 66005–1-Ig, 1:300).

### 2.9 In vitro anti-inflammatory effect

For all the cell experiments, RAW264.7, CaCO_2_ and NCM460 cells were cultured with MSH@E extract liquid. Typically, MSH@E microcapsules were incubated with RPMI 1640 medium or DMEM medium for 72 h. After that, the MSH@E extract liquid was obtained by collecting the supernatant by centrifugation and then filtered. After being seeded in six-well plate overnight, the cells were co-cultured with LPS (1 μg/mL) and then incubated with MSH@E extract liquid (322 μg/mL, equivalent to 20 μg/mL His-MANF protein) for 24 h. Subsequently, cells were lysed by RIPA lysis buffer (Beyotime, P0013B), and the protein expression of IL-6 (Sigma-Aldrich, Ab 1839, 1:1000), IL-1β (Abcam, Ab 9722, 1:1000), NF-κB p65 (EMD Millipore, 06–418, 1:1000), and β-actin (Affinity, T 0022, 1:1000) was analyzed by western blot (WB) and quantitative real-time PCR (details in the supporting information). The resulting bands were analyzed quantitatively by using Image J software. Additionally, the level of myeloperoxidase (MPO) activity (Boster, EK0943; Reed Biotech, RE3101H), IL-6 (Boster, EK0411; Reed Biotech, RE3186H), IL-1β (Boster, EK0394; Reed Biotech, RE1074H) and TNF-α (Boster, EK0527; Reed Biotech, RE1060H) in the cell supernatant was measured by ELISA.

### 2.10 In vivo pharmacodynamics of MSH@E against colitis

Colitis mice were randomly divided into 7 groups: PBS—colitis mice orally treated with PBS, 5-ASA—colitis mice orally treated with 5-ASA (1 mg/kg), SH@E—colitis mice orally treated with SH@E (16 mg/kg), MS@E—colitis mice orally treated with MS@E (equivalent to 1 mg/kg of His-MANF), His-MANF (i.v.)—colitis mice intravenously injected with His-MANF (1 mg/kg), MSH@E—colitis mice orally treated with MSH@E (equivalent to 1 mg/kg of His-MANF), and 1/2 MSH@E—colitis mice orally treated with half a dose of MSH@E group (equivalent to 0.5 mg/kg of His-MANF). Drug administration was performed once per day from day 4 to day 7. The body weight and food intake of the mice were recorded daily. The disease activity index (DAI) was calculated by summing the scores for stool consistency (0–3), rectal bleeding (0–3), and weight loss (0–4) (Huang et al., 2022). On the 10th day, the mice were killed. The weight and length of the colons were measured. Colon tissues were further sectioned for HE staining. The serum of mice was collected for further measurement of MPO activity (Boster, EK0943), IL-6 (Boster, EK0411), IL-1β (Boster, EK0394) and TNF-α (Boster, EK0527) by ELISA following the manufacturer's instructions.

### 2.11 Statistical analysis

Statistical analysis was performed by GraphPad Prism 9.0. Quantitative results were presented as mean values with standard deviation (SD) from three repeated experiments. The two-tailed and unpaired *t*-test was used to compare two experimental groups. *p* < 0.05 was considered statistically significant.

## 3 Results

### 3.1 Preparation and characterization of MSH@E microcapsules

**Fig. 1:**
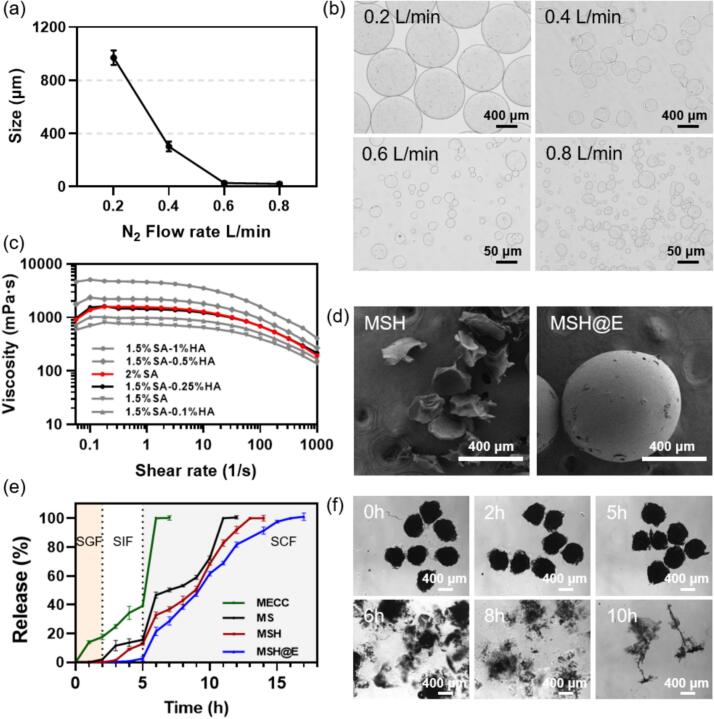
Characterization of MSH@E microcapsules. (a) The size change of SA microspheres with gas flow rate. (b) Optical images of MSH microcapsules prepared with different gas flow rate. (c) Rheological properties of SA and HA in different proportions. (d) SEM images of MSH and MSH@E microcapsules. (e) Release curves and (f) optical microscopy images of His-MANF in a stimulated GIT environment.

SA hydrogel microspheres with different particle sizes were successfully prepared by gas flow shear equipment (Scheme 1). The preparation speed of the microcapsules can be tuned via the flow rate of the SA solution. More importantly, the size of the prepared microcapsules could be adjusted by changing the gas flow rate. The diagram of size with flow rate is shown in Fig. 1a. Specifically, the hydrogel microcapsules with a size of 26 μm were observed at 0.6 L/min, while the hydrogel microcapsules with a size of 21 μm were observed at 0.8 L/min. The continuous increase of the gas flow rate in this device had a slight effect on the particle size. Moreover, Eudragit S100 was difficult to coat the microsphere with a large particle size, and therefore 0.6 L/min was used to prepare SA hydrogel microcapsules in the subsequent experiments. The morphology of the microcapsules observed under an optical microscope showed that the prepared SA hydrogel microcapsules were spherical or quasi-spherical. As gas flow rate increased, the particle size of the microcapsules showed a decreasing trend, and the shape of the microcapsules became less uniform (Fig. 1b; Fig. S1).

Since the purification of His-MANF is complex, BSA was selected to explore the optimal amount of protein, SA and CaCl_2_ considering both LE and EE. As SA concentration increased, EE increased and LE decreased. It was noteworthy that both LE and EE increased when the concentration of CaCl_2_ was 10 %, which might be ascribed to the high crosslinking of SA and CaCl_2_ making it much easier to encapsulate proteins (Feng et al., 2023). As the concentration of BSA increased, LE increased and EE decreased. As the volume ratio of SA and CaCl_2_ increased, both LE and EE increased simultaneously. Based on the above results, His-MANF-loaded SA hydrogel microcapsules (MS) were prepared by using 2 mg/mL BSA, 2 % *w*/*v* sodium alginate, 2 % w/v calcium chloride concentration, and the volume ratio (2 % w/v SA: 2 % w/v CaCl_2_) of 3:1 as the starting materials (Table S2).

Considering HA could specifically bind to the CD44 receptor of inflamed macrophages, we intended to mix it with SA in the above preparation process to get multi-functional microcapsules. However, it is difficult to obtain stable hydrogel microcapsules by adding HA directly to 2 % w/v SA. Rheological behavior is an important parameter for polysaccharides and is affected by the polysaccharide type and concentration, and therefore the rotary rheometer behavior of the mixture of HA and SA with different ratios was measured to explore the optimal ratio of HA to SA. The results showed that the rheological behavior of 1.5 % w/v SA mixed with 0.25 % w/v HA was most similar to that of 2 % w/v SA, so this formulation was selected for the preparation of SA/HA mixed hydrogel microcapsules (Fig. 1c). Besides, Eudragit S 100 was also coated on the His-MANF encapsulated SA/HA hydrogel (MSH) microcapsules to achieve higher bioavailability. After Eudragit S100 coating, the size increased, and the initial rough surface became smooth under SEM observation (Fig. 1d), indicating that Eudragit S 100 was successfully coated onto the surface of MSH microcapsules.

The LE and EE of MSH@E microcapsules examined by Bradford method indicated that the LE and EE were 6.14 % and 80.86 %, respectively. After oral administration, His-MANF will enter the digestive tract. However, the pH value of different parts of the digestive tract is greatly different. We conducted in vitro drug release experiment of MSH@E microcapsules in a simulated upper gastrointestinal tract (GIT) buffer (Fig. 1e). It was found that His-MANF was barely released when exposed 2 h in SGF (pH 1.2), and only approximately 5 % of the protein was released in the SIF (pH 6.8). When MSH@E microcapsules were transferred to the SCF (pH 7.4), His-MANF was rapidly released and released completely within 17 h. We also encapsulated His-MANF protein directly in enteric-coated capsules (MECC), the results showed a very fast release in SIF and SCF, suggesting the SA/HA hybrid hydrogel sustains the release of His-MANF protein. The morphology of MSH@E microcapsules was also monitored under an optical microscope, and the results exhibited similar change to the release profiles. As shown in Fig. 1f, the morphology of the microcapsules kept almost the same in SGF and SIF. While at high pH condition, surface Eudragit S100 dissolved and the microcapsules collapsed quickly due to the swelling and erosion of SA. The released His-MANF protein was also characterized by SDS-PAGE (Fig. S2). Compared with the purified His-MANF protein (land 4), the His-MANF protein exposed to SGF precipitated and degraded quickly, resulting in the disappearance of the band (lane 3). To the contrary, His-MANF protein released from MSH@E after exposed to the simulated GIT still showed a clear band.

### 3.2 Biocompatibility evaluation of MSH@E microcapsules

**Fig. 2:**
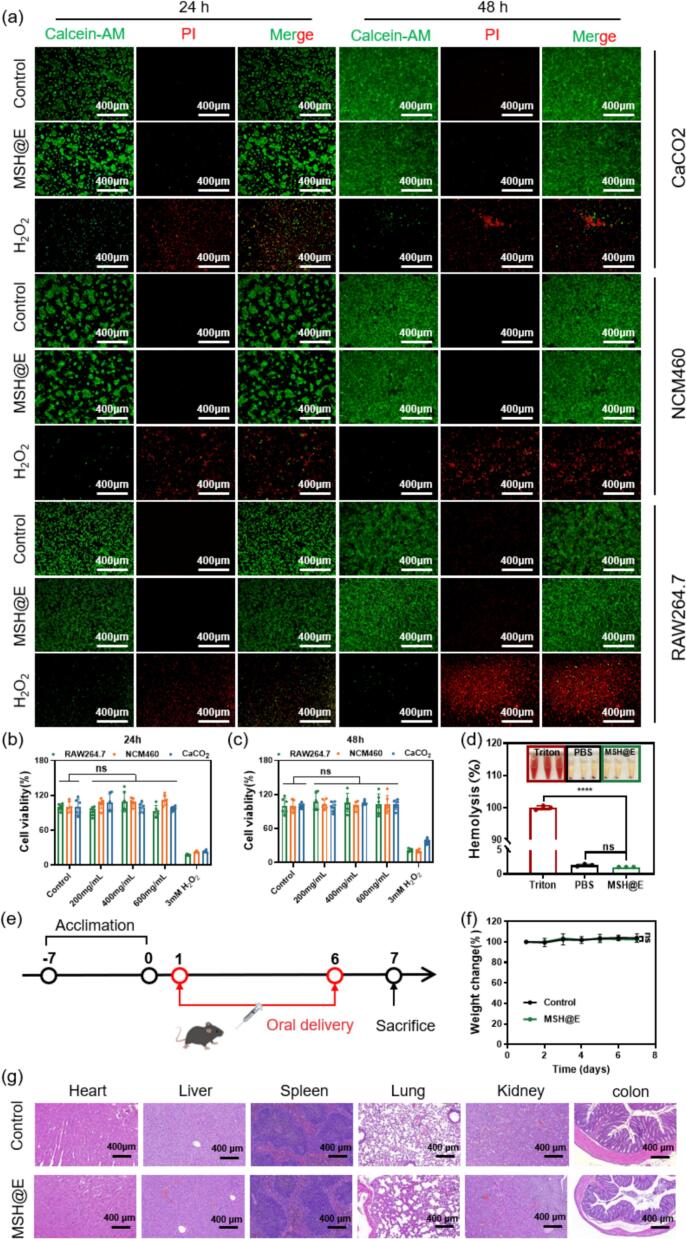
Biocompatibility evaluation of MSH@E microcapsules. CaCO_2_, NCM460 and RAW 264.7 cells were used to assess the cytotoxicity of microcapsules. (a) Calcein AM/PI staining. (b-c) MTT assay. (d) Hemolysis test. The inset is the corresponding digital images. (e) Experimental protocol of the in vivo biocompatibility evaluation. C57BL/6 J mice were orally treated with PBS or MSH@E (His-MANF: 15 mg/kg/day) for 6 days. (f) Body weight change. (g) HE staining images of major organ tissues. Data are mean ± S.E.M. (ns: not significant, ^⁎⁎⁎⁎^*p* < 0.0001).

It is important to evaluate the biocompatibility of the drug delivery system before in vivo application. CaCO_2_, NCM460 and RAW 264.7 cells were used to assess the cytotoxicity of microcapsules. Calcein AM/PI staining results (Fig. 2a) indicated that the number of death cells was very limited even when the co-incubation time reached 48 h, suggesting MSH@E microcapsules had low cytotoxicity. Then, MTT assay was also used to evaluate the cytotoxicity of MSH@E microcapsules. According to the MTT assay results, MSH@E microcapsules didn't show any significant cytotoxicity to CaCO_2_, NCM460 and RAW 264.7 cells compared with the control group after 48 h of treatment (Fig. 2b-c).

For the hemolysis test, red blood cells were exposed to MSH@E microcapsules. The digital images showed that there was almost no hemolysis in the supernatant of the MSH@E microcapsules treated group. The transmittance of the supernatant of red blood cells after MSH@E microcapsules treatment was <2 % (Fig. 2d), suggesting the hemolytic ability of the MSH@E microcapsules was negligible.

Histology changes are an important aspect of in vivo toxicity. For in vivo acute toxicity evaluation, healthy mice were divided into two groups and orally administrated with PBS and MSH@E microcapsules at a dosage form of 750 mg/kg/day (equivalent to His-MANF 50 mg/kg/day) for 6 consecutive days, respectively. During the process, body weight was recorded daily. As shown in Fig. 2f, there is no obvious difference between PBS group and MSH@E group. On the 7th day, mice were killed, and major organs (heart, liver, spleen, lung, kidney, and colon) of mice were taken for H&E staining. Histological analysis showed that there were no obvious lesions in the heart, liver, spleen, lung, kidney, colon and other parts of the mice after oral daily administration of MSH@E for 6 days (Fig. 2g). These results indicated that MSH@E has good biocompatibility and almost no toxic side effects.

### 3.3 Biodistribution of MSH@E microcapsules

**Fig. 3:**
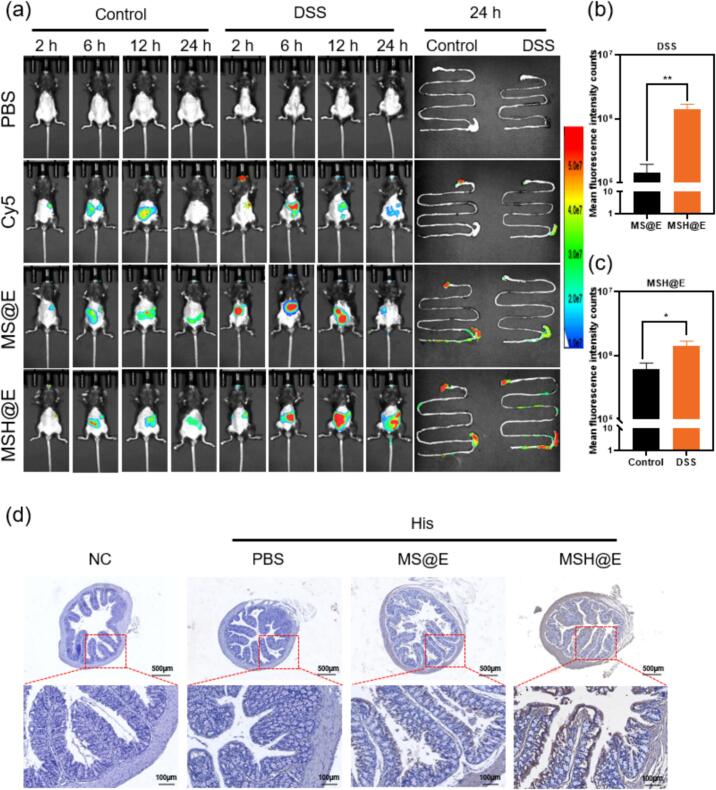
Biodistribution of MSH@E microcapsules after oral administration. (a) In vivo fluorescent imaging of healthy control mice and DSS colitis mice after being orally treated with PBS, Cy 5, MS@E, and MSH@E microcapsules. The right were the corresponding GIT fluorescent images. (b) The mean fluorescence intensity of GIT of MS@E and MSH@*E*-treated DSS colitis mice. (c) The mean fluorescence intensity of GIT of MSH@E treated DSS colitis mice and MSH@E-treated healthy control mice. (d) Immunohistochemistry staining of colon section from healthy mice and colitis mice after administration with PBS, MS@E and MSH@E.

To examine the biodistribution of MSH@E in both healthy mice and DSS-induced colitis mice by in vivo fluorescent imaging, His-MANF protein was first labelled by Cy 5. As expected, it was found that the fluorescence of MSH@E-treated colitis mice was stronger than that of MS@E treated colitis mice (Fig. 3a) and the fluorescence of MSH@E-treated colitis mice was stronger than that of MSH@E-treated healthy mice. After 24 h, heart, liver, spleen, lung, and kidney were harvested and the results showed that only a few visible fluoresce, suggesting that very few His-MANF proteins accumulated in these organs (Fig. S3). As shown in the ex vivo GIT images, the strong fluorescence signal also evidenced that His-MANF protein mainly accumulated in GIT via oral administration. Consistent with the in vivo results, the average fluorescence intensity of GIT in MSH@E-treated colitis mice was stronger than that of MS@E-treated colitis mice (Fig. 3b), the average fluorescence intensity of MSH@E- treated colitis mice was higher than that of MSH@E-treated healthy mice (Fig. 3c). In addition, the colon section was also immunohistochemically stained with His tag antibody to evident whether exogenous His-MANF protein was delivered to the colon tissue. As shown in Fig. 3d, MANF proteins with His tags were found in the colon tissue of mice in both MSH@E and MS@E group, and MSH@E-treated colitis mice show greater accumulation than MS@E-treated colitis mice, which demonstrated that HA enhanced the localization within the inflamed colon.

### 3.4 In vitro anti-inflammation activity

**Fig. 4:**
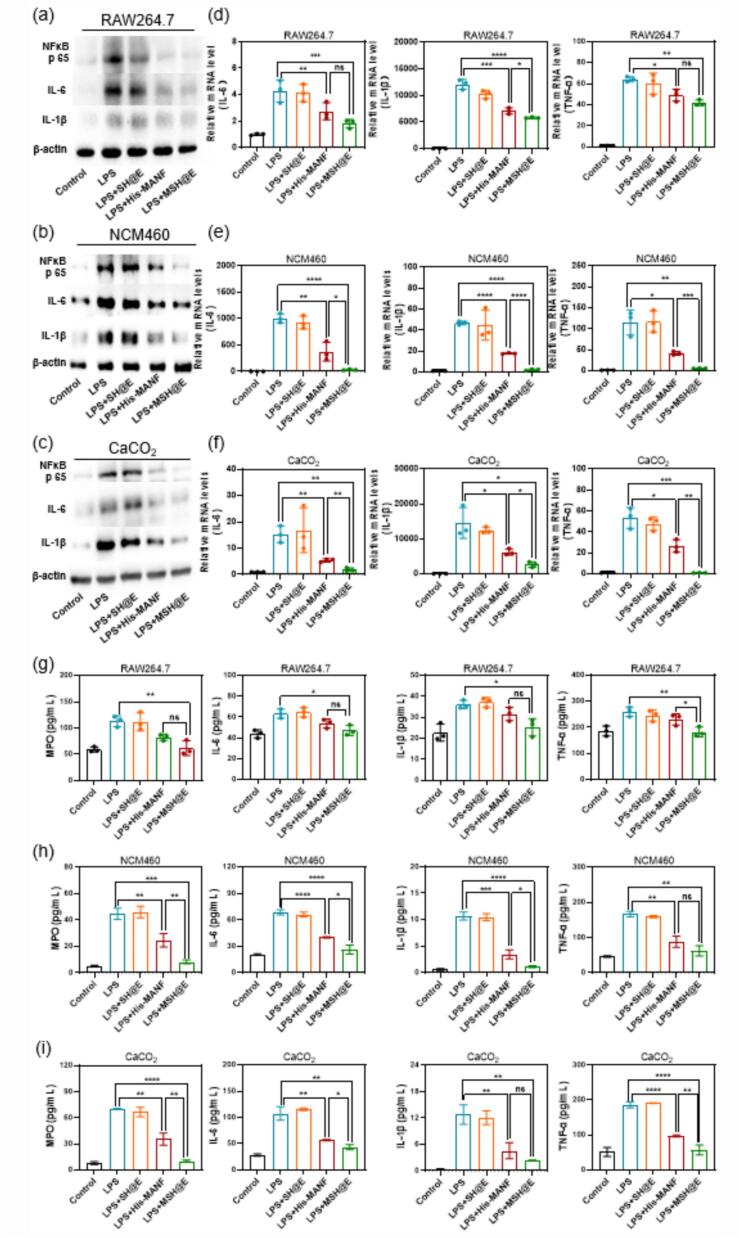
In vitro anti-inflammatory effect of MSH@E microcapsules. Expression of inflammatory factors (NF-κB p65, IL-6, IL-1β, TNF-α) in LPS-induced RAW 264.7, CaCO_2_ and NCM460 cells with different treatments determined by WB, qPCR and ELISA. (a-c) WB analysis of NF-κB p65, IL-6 and IL-1β level. (d-f) qPCR analysis of the expression of IL-6, TNF-α and IL-1β. (g-i) The expression level of IL-6, IL-1β, TNF-α and MPO in LPS-induced RAW 264.7 cell supernatant. All the data were represented as means ± SD. (ns: not significant, ^⁎^*p* < 0.05, ^⁎⁎^*p* < 0.01, ^⁎⁎⁎^*p* < 0.001, ^⁎⁎⁎⁎^*p* < 0.0001).

To evaluate the anti-inflammation effect of MSH@E microcapsules, LPS-activated RAW264.7, NCM460 and CaCO_2_ cells were incubated with PBS, His-MANF, SH@E and MSH@E microcapsules and then determined by WB, qPCR and ELISA. WB experiment results demonstrated the levels of NF-κB p65, IL-6 and IL-1β increased after LPS activation. After His-MANF and MSH@E treatment, the levels of NF-κB p65, IL-6, and IL-1β decreased (Fig. 4a-c). The mRNA levels of IL-6, IL-1β, and TNF-α were significantly increased compared with the control group after LPS activation. His-MANF and MSH@E microcapsules exposure resulted in downregulation of the above proinflammatory genes. Meanwhile, blank SH@E also mildly decreased the mRNA level of IL-6, IL-1β, and TNF-α, which may be ascribed to the anti-inflammatory effects of SA and HA (Litwiniuk et al., 2016; Yamamoto et al., 2013) (Fig. S4). We also measured levels of IL-6, IL-1β, TNF-α, and MPO in LPS-induced RAW 264.7 cell supernatant by using ELISA. As shown in (Fig. 4g-i), His-MANF and MSH@E also showed a good anti-inflammatory effect, which was consistent with WB and qPCR results.

### 3.5 MSH@E microcapsules alleviated DSS-induced colitis

**Fig. 5:**
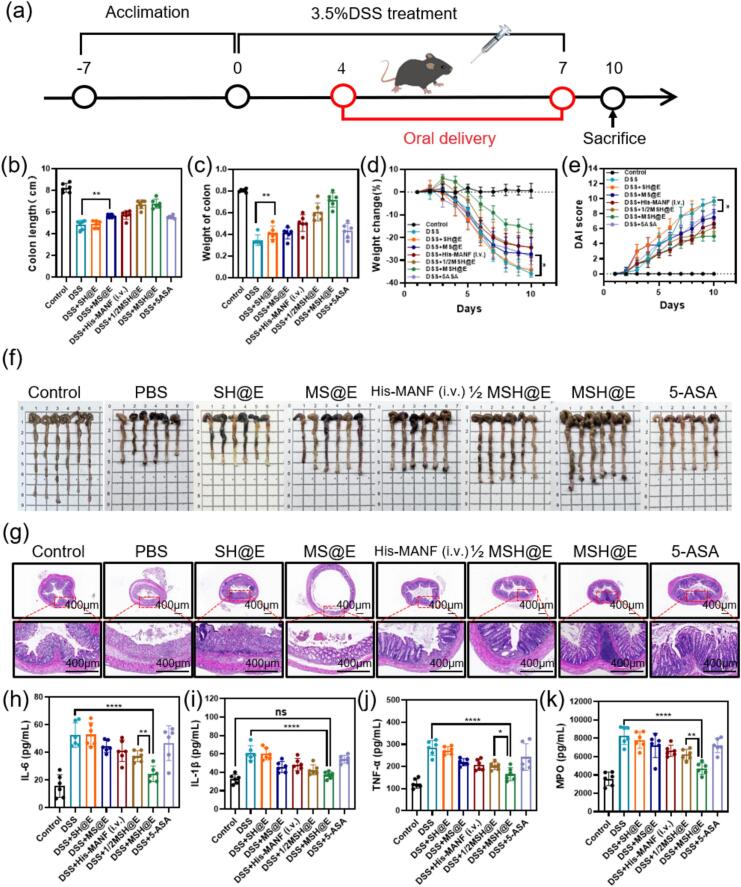
In vivo therapeutic effects of MSH@E against UC. (a) Schematic diagram of the therapy of colitis mice. C57BL/6 J mice were given drinking water containing DSS (3.5 %, *w*/*v*) for 7 days and received drug treatment from day 4 to day 7. (b) Weight of colon. (c) Colon lengths. (d) Body weights. (e) DAI scores and (f) Digital photos of colons. (g) HE staining images of colon sections. (h-k) The level of IL-6 (h), IL-1β (i), TNF-α (j) and MPO (k) in serum. Data are mean ± S.E.M. (*n* = 6; ns: not significant, ^⁎^*p* < 0.05, ^⁎⁎^*p* < 0.01, ^⁎⁎⁎^*p* < 0.001, ^⁎⁎⁎⁎^*p* < 0.0001).

A mouse colitis model induced by DSS was established to evaluate the therapeutic effect of MSH@E microcapsules (Lee et al., 2020). As shown in Fig. 5a, water containing DSS (3.5 %, w/v) was given to mice for 7 days and drug administration was performed from day 4 to 7. During the whole process, each mouse's body weight was recorded and analyzed (Fig. 5d). The body weight of healthy mice with normal feeding had a slight increase, while mice in DSS group treated with PBS lost nearly 40 % of their body weight. The weight loss of the mice in 5-ASA, His-MANF (i.v.), MS@E and MSH@E groups was alleviated. Especially, MSH@E microcapsules had the best therapeutic effect against body weight loss. Similarly, the disease activity index (DAI) scores of the DSS treated with PBS were obviously higher than those of healthy mice. Mice in DSS group treated with 5-ASA, His-MANF (i.v.), MS@E and MSH@E microcapsules had lower DAI scores than mice in the DSS treated with PBS (Fig. 5e; Fig. S5). On the 10th day, all the mice were killed. The colons were collected and imaged (Fig. 5f). The statistics of colon weight and colon length (Fig. 5b and c) showed that treatment with MSH@E microcapsules reduced shortening of colon length and enlargement of the spleen.

Moreover, colon tissues of mice were stained by HE to examine the histological changes to evaluate the therapeutic effects of MSH@E against UC. As shown in Fig. 5g, HE-stained colon sections of mice in DSS group treated with PBS had obvious neutrophils infiltration, extensive mucosa damage, crypts disappearance, goblet cells depletion and other inflammatory signs. After 5-ASA, His-MANF (i.v.), MS@E and MSH@E microcapsules treatment, inflammatory signs were significantly reduced.

MPO, a biomarker for neutrophil infiltration, increased significantly in the blood serum of the colitis mice (Fig. 5k), indicating severe neutrophils infiltration in the colon. MSH@E dramatically decreased the MPO level. Meanwhile, the levels of IL-6, IL-1β and TNF-α of mice in the MSH@E group were decreased compared to those of mice in DSS group treated with PBS (Fig. 5h-j), suggesting the inflammation was suppressed by MSH@E microcapsules.

### 3.6 MSH@E microcapsules regulated the gut microbiota

**Fig. 6:**
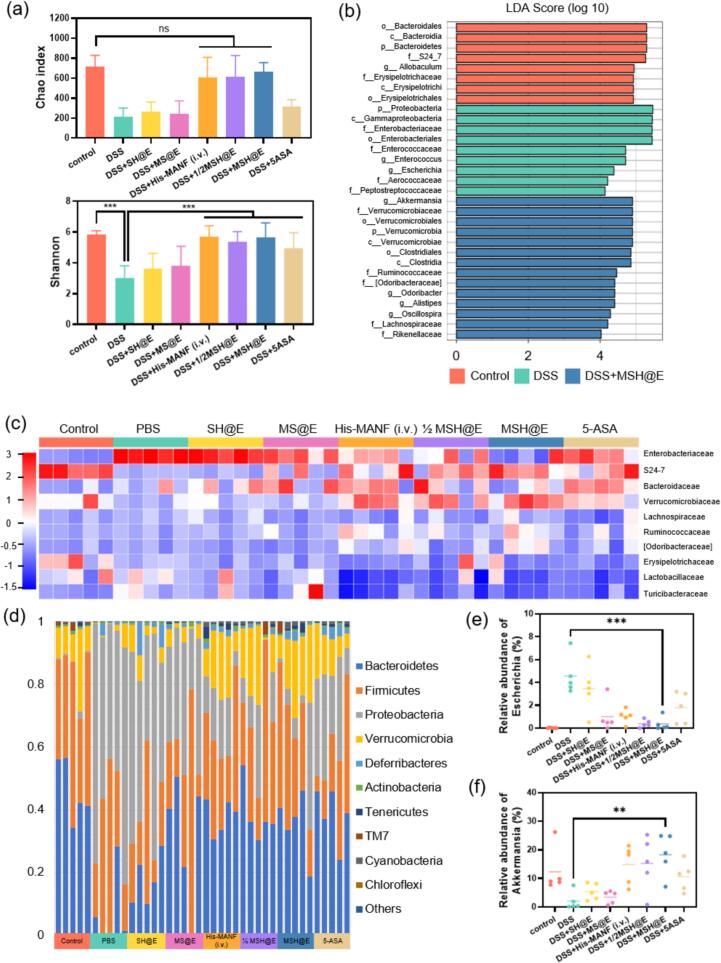
16S ribosomal RNA (rRNA) sequencing analysis of intestinal flora after different treatment. (a) Chao and Shannon index. (b) Linear discriminant analysis (LDA), threshold: 4. (c) Heatmap. (d) Community histogram. (e-f) Relative abundance of Escherichia-Shigella (e) and Akkermansia (f). *n* = 5, ns: not significant, ^⁎^*p* < 0.05, ^⁎⁎^*p* < 0.01, ^⁎⁎⁎^*p* < 0.001.

The intestinal microbiota is closely associated with gut health and inflammatory bowel disease (IBD) (Lee et al., 2020). The abundance of gut flora were assessed using 16S rRNA gene sequencing after different treatments. The abundance of the bacterial community of colitis mice was obviously changed compared with the healthy mice (Fig. S6). Intravenous His-MANF and oral MSH@E, 5-ASA significantly increased Chao 1 and Shannon diversity indices and improved species richness and microbial community diversity (Fig. 6a). Differential abundance taxa were determined to be differential abundance taxa using linear discriminant analysis (LDA) analysis. LDA scores showed the effects of dominant groups and different taxonomic levels including from phylum to genus (Figs. 6b and S6). The general Enterobacteriaceae composition at the family and phylum level for each sample was displayed in a heatmap and bar chart, respectively (Figs. 6c and d). Escherichia -Shigella, a highly virulent strain of Proteobacteria, has been reported to be significantly increased in mice with inflammatory bowel disease (Fan et al., 2021). Akkermansia, belonging to the Verrucomicrobia, plays a crucial role in safeguarding the integrity of the intestinal barrier and is considered a beneficial probiotic (Plovier et al., 2017). MSH@E microcapsules decreased the relative abundance of Escherichia-Shigella and increased the relative abundance of Acromancia (Fig. 6e and f). The abundance of Actinobacteria was found to be diminished in the DSS group, whereas the abundance of Proteobacteria was raised (Fig. S6), which is consistent with the flora composition of patients with IBD (Schirmer et al., 2019). The above results indicated that MSH@E microcapsules could help UC mice restore dysbiosis.

## 4 Discussion

Protein-based biologics play an important role in modern medicine for their low side effects. For IBD, oral administration is an ideal administration stratagem due to its convenience, reduction of the generation of anti-drug antibodies, and rapid accumulation in the inflammatory lesion area. However, the studies about His-MANF protein are intravenous injection or local administration. Therefore, it is of great significance to develop an oral delivery system for His-MANF protein and study the efficacy in alleviating IBD. Our study is novel for the combination of oral and colon-targeted delivery His-MANF protein.

Both SA and HA are natural polymers and have good biocompatibility. The combination of HA with SA forms a biomimetic hybrid hydrogel microcapsule, which is a kind of ideal drug carrier. MSH hydrogel microcapsules were first prepared by gas flow shear equipment. This method can adjust the preparation speed and size of the microcapsules easily. Additionally, the preparation process was not chemically addictive, and the operation condition was mild, which is superior to the microfluidic method. This mild prepare process is very beneficial for protein loading. In addition, the preparation process combined with gas shear technology, chemical cross-linking technology, and emulsification coating method has many advantages, including easy operation, high efficiency, and great feasibility, which makes it very suitable in clinical transformation.

In the MSH@E delivery system, His-MANF protein has an isoelectric point of approximately 8.5, resulting in a positive charge under neutral conditions. Meanwhile, both SA and HA carry negative charges, so there will be an electrostatic interaction among them. This interaction makes His-MANF encapsulated into the hybrid microcapsules much more firmly, which can also be reflected by the His-MANF release behavior between MS and MSH. As shown in Fig. 1e, His-MANF loaded in MSH@E microcapsules showed a slow release compared with MS and MSH. The release of MSH@E was hindered in low pH due to the out ES 100 layer. ES 100 only dissolves when pH greater than 7. After ES 100 dissolved, the alginate microcapsules became exposed to the colonic environment, and then the encapsulated His-MANF protein was released through SA swelling and erosion. The delivery system exhibits a controlled and sustained release behavior due to the formation of SA hydrogel and the coating of ES 100. The accumulative drug release amount was less than 5 % after exposure in SGF for 2 h and SIF for 3 h. MSH@E microcapsules showed sustained release for 17 h, which is longer than MECC and MSH microcapsules. It was concluded that His-MANF protein could be successfully delivered to the colon, which was also evident by the in vivo experiments. What's more, His-MANF released from MSH@E microcapsules after exposure in SGF for 2 h and SIF for 3 h in SDS-PAGE pattern also showed a clear band, suggesting the MSH@E microcapsules protect His-MANF from degradation in the harsh upper GIT environment.

For the in vitro experiments, RAW264.7 and CaCO_2_, NCM460 cells were cultured with MSH@E extract liquid. It was difficult to quantify accurately to a low concentration scale of MSH@E due to the micron scale size range. Therefore, we first incubated MSH@E in cell culture medium for enough time to make all the components to be extracted thoroughly. Then the extract liquid was diluted with fresh culture medium according to the specific requirements. When MSH@E exposed to cell culture medium, the outer ES100 layer dissolved, and then alginate swelled and finally formed a colloid solution. Therefore, it is believed that this extract will have the same effect as MSH@E. This method was also found in previous reports (Zhu et al., 2023).

In the bio distribution study, MSH@*E*-treated colitis mice showed much stronger fluorescence intensity than MS@E-treated colitis mice, and MSH@E showed a longer retention time in the colitis colon than free Cy 5. In addition, MSH@E showed stronger fluorescence in the DSS group than that in the healthy control group. All the phenomena suggested MSH@E had a preferential interaction with inflamed colon tissue. The immunohistochemistry staining further indicated that His-MANF protein was accumulated in the inflamed mucosae and epithelium. As shown in Scheme 1, the outer layer of Eudragit S100 dissolved in the intestine after MSH@E oral treatment. Then, MSH hydrogel microspheres swelled in the colon and adhered to the colitis inflammation area due to the electrostatic interaction and the binding effect of CD 44 on the inflamed endothelial cells with HA. As time prolonged, His-MANF, HA, and SA dissolved and crossed the endothelial barriers due to the enhanced permeability of endothelial cells and the damage of the mucus barriers. We guessed that there might be a weak interaction (such as hydrogen bonds) between HA and His-MANF, and therefore the uptake of His-MANF by inflamed macrophages could be enhanced via CD44 medicated endocytosis. This could also explain why oral MSH@E microcapsules had a better anti-inflammation effect than His-MANF (i.v.) group even the dose of the His-MANF in MSH@E microcapsules (500 μg/kg) was only half of the His-MANF intravenously injected (1 mg/kg).

In the pharmacodynamics study, it was very exciting that MSH@E showed more effectiveness than 5-ASA in all the therapeutic results, and orally delivering MSH@E with a His-MANF dose of 500 μg/kg even gained much better effect than intravenous injection of His-MANF (1000 μg/kg), suggesting the effectiveness of the oral colon-targeted delivery system. Compared with mice in the healthy control group, mice in the MSH@E group showed almost no significant difference (Fig. 5). Taken together, MSH@E showed the best therapeutic effect against UC among the treated DSS groups.

The intestinal microbiota is very important for maintaining intestinal homeostasis(Chen and Nuñez, 2010). This study shows that there are a large number of potential infectious Escherichia-Shigella in the intestinal microbiota of mice induced by DSS, which should partially contribute to the development of colitis. MSH@E treatment transformed the characteristics of the intestinal microbiota from a dysregulated state to a normal state.

Our previous studies had shown that His-MANF could inhibit LPS-induced NF-κB activation in lung tissues and macrophages, thereby reducing the expression of NF-κB target genes, such as TNF-α, IL-1β, and other pro-inflammatory factors. As a result, the number of M1 proinflammatory macrophages decreased and the number of M2 anti-inflammatory macrophages increased (Shen et al., 2022). Recent studies have found that MANF can reduce the release of inflammatory factors by downregulating the NF-κB pathway in pro-inflammatory macrophages and thereby reducing inflammation. In addition, MANF also decreased the levels of inflammatory factors by promoting pro-inflammatory macrophage apoptosis (Wang et al., 2023). Therefore, the released His-MANF from MSH@E microcapsules may suppress M1 macrophage polarization and promote M2 macrophage polarization by inhibiting NF-κB activation to alleviate inflammation. Additionally, MSH@E microcapsules could increase the relative abundance of beneficial probiotics, which was further beneficial for the alleviation of colitis.

Although we demonstrated the effectiveness of the MSH@E oral colon-targeted delivery system, we used only one animal model of DSS induced colitis. It is also necessary to research the long-term treatment and recurrence of IBD since it's a chronic disease with recurrence. It may be more convincing to evaluate the efficacy on more diverse and accurate animal colitis models in future studies.

## 5 Conclusion

In summary, we have developed a new oral His-MANF colon-targeted delivery system to the inflammatory colon for the alleviation of colitis. The nature polymer sodium alginate hydrogel's chemical cross-linking properties with calcium ions successfully encapsulated the protein, while the hyaluronic acid binds with CD44 made MSH tend to attach to the site of inflammation, and the polymer Eudragit S 100 further protected the encapsulated His-MANF and increased tolerance of His-MANF to harsh conditions during gastrointestinal transport. The system reduces systemic exposure and prolongs the residence time of His-MANF, thereby maximizing the bioavailability of the drug. In addition, MSH@E microcapsules can regulate inflammation by inhibiting the secretion of pro-inflammatory cytokines, and modulation of gut microecology in vivo. This His-MANF oral formulation even enhanced colitis efficacy than His-MANF intravenous injection, suggesting a breakthrough from intravenous injection to oral administration, which would accelerate its translational medicine applications.

## Funding

This work was funded by University Excellent Scientific Research and Innovation Team of Anhui Province (Grant No. 2022AH010046 to Y.-X. S.) and Research Fund of Anhui Institute of translational medicine (Grant No. 2022zhyx-B09 to M.-M. S.) and Anhui Provincial Colleges and Universities Scientific Research Project (Grant No. 2024AH050690 to M-M Song).

## Ethical approval

The animal experiments followed the guidelines of the Institutional Animal Care and Use Committee of Anhui Medical University (Agreement numbers: LLSC20231957).

## Consent for publication

No human research participants or images were involved in this study, so no informed consent was required for publication.

## CRediT authorship contribution statement

**Jie Zhou:** Writing – original draft, Visualization, Validation, Software, Methodology, Investigation, Formal analysis, Data curation, Conceptualization. **Tian-Le Li:** Writing – review & editing, Validation, Software, Methodology, Investigation, Conceptualization. **Bo Wei:** Visualization, Validation, Software, Resources, Methodology, Investigation. **Yue-Feng Ruan:** Visualization, Validation, Methodology, Investigation. **Ye-Qin Wang:** Visualization, Validation, Methodology, Investigation. **Jiao-Yan Liu:** Methodology, Formal analysis, Conceptualization. **Meng-Meng Song:** Writing – review & editing, Supervision, Resources, Project administration, Methodology, Funding acquisition, Formal analysis, Conceptualization. **Yu-Xian Shen:** Writing – review & editing, Supervision, Resources, Project administration, Methodology, Funding acquisition, Formal analysis, Conceptualization.

## Declaration of competing interest

The authors declare no conflict of interest.

## Data Availability

Data will be made available on request.

## Appendix A Supplementary data

Supplementary material Image 1
