# Supplementary material for: Oral colon-targeted delivery of recombinant human MANF for alleviation of ulcerative colitis
Source: Int J Pharm X. 2025 Feb 26;9:100320. doi: 10.1016/j.ijpx.2025.100320 (PMC11925120; doi:10.1016/j.ijpx.2025.100320)
Supplement: Supplementary file 1 — Supplementary material [file mmc1.docx]

**Supplementary materials:**

**Oral Colon-Targeted Delivery of Recombinant Human MANF for Alleviation of Ulcerative Colitis**

Jie-Zhou, Tian-Le Li, Bo-Wei, Yue-Feng Ruan, Ye-Qin Wang, Meng-Meng Song^*^, Yu-Xian Shen^*^

School of Basic Medical Sciences, Anhui Medical University, 81 Meishan Road, 230032 Hefei, Anhui, PR China.

**1. MTT assay**

For MTT assay, RAW 246.7, NCM460 and CaCO_2_ cells were seeded in 96-well plates (1×10^4^/ well). After 12 h, MSH@E extract was introduced to the medium and co-incubated for 24 h and 48 h, respectively. Then, 10 μl MTT (5 mg/mL) were introduced and incubated for 4 h at 37 °C. After that, the MTT formazan was dissolved in DMSO and A_490 nm_ was measured in a microplate reader (Biotek; Synergy H1). Finally, the cell viability was calculated as:

Cell viability (%)= (A _tested cells_/A _control cells_) × 100%.

**2. Hemolysis assays**

For hemolysis assay, MSH@E microcapsules were incubated with PBS for 72 h. After that, the MSH@E extract liquid was obtained by collecting the supernatant by centrifugation and then filtered. The blood samples were centrifuged at 1700 g/min. The supernatant was removed by aspiration and 2 mL of PBS was added. After the washing step repeated for 3 times, the remaining pellet was diluted 2:100 in PBS. Then 50 μL MSH@E extract, water or TritonX-100 (0.1 % v/v) were mixed with 2% erythrocyte suspension. Then the mixture was incu­bated for 2 h at 37 ◦C. after incubation, the mixture was centrifuged at 10,000 rpm for 10 min, and the absorbance of supernatant was measured at 545 nm in a microplate reader (Biotek; Synergy H1). The hemolysis (%) was calculated as:

Hemolysis (%) = (A _test samples_-A_negative control_/A_positive control_-A_negative control_) × 100%

**3. Calcein AM and propidium iodide (PI) staining**

For live/dead cell staining, RAW 246.7, NCM460 and CaCO_2_ cells were seeded in 24-well plates at a density of 5×10^4^ cell per well. After 12 h, MSH@E extract was added to the culture medium for 24 h and 48 h, respectively. Then the cells were stained with Calcein AM and propidium iodide (PI) for 30 min and then imaged with fluorescent microscopy (OLYMPUS; IX73).

**4. Western blot**

Cells were placed on ice, washed 3 times with PBS and lysed by adding RIPA lysate containing protease inhibitor. After sufficient lysis, the precipitate was removed by centrifugation in an ultracentrifuge at 10000 rpm at 4 ℃, and the supernatant was heated with 5×SB in a water bath for 10 min after which it was sampled on 12% SDS-PAGE. Subsequently, the protein was transferred to the PVDF membrane. The membranes were closed with skimmed milk, incubated with primary and secondary antibodies, and finally tested for immunoreactivity by the ECL blotting detection system.

**5. HE Staining**

The tissue samples were fixed in 4% paraformaldehyde. After the dehydration in ethanol, the tissue was embedded in paraffin then sectioned with a thickness of 4 µm. The sections were first deparaffinized in different concentrations of alcohol (100%, 90%, 80%, 70%) and finally placed in distilled water for 5 min at each step. Sections were stained in hematoxylin for 1 min and then rinsed in water for 10 min. Finally, the sections were stained in eosin solution for 3 min and sealed for observation.

1. **Immunohistochemistry Staining**

The tissue samples were fixed in 4% paraformaldehyde. After the dehydration in ethanol, the tissue was embedded in paraffin then sectioned with a thickness of 4 µm. Paraffin-embedded colon sections (4 μm) were used for IHC of his. In brief, sections were incubated with primary antibodies overnight at 4°C. After washing in PBS, the sections were incubated with the appropriate biotinylated secondary antibody for 1 h at 37 °C, which followed by incubation with horseradish peroxidase conjugated streptavidin for 15 min at 37°C. Then, the sections were counterstained with hematoxylin. Images were acquired using Olympus Microscope BX53 and cell Sens Standard software.

1. **Quantitative real-time PCR (qPCR)**

Trizol reagent (Invitrogen, USA) was used for extracting total RNA from colon tissues according to the manufacturer’s protocol, and reverse transcription was performed at 37°C for 30 min using PrimeScriptTM RT Master Mix (Takara, Japan).

Table S1 Primers sequences for qPCR

| Gene | Forward | Reverse |
| --- | --- | --- |
| GAPDH | AGGTCGGTGTGAACGGATTTG | GGGGTCGTTGATGGCAACA |
| IL-6 | CTGCAAGAGACTTCCATCCAG | AGTGGTATAGACAGGTCTGTTGG |
| IL-1β | GAAATGCCACCTTTTGACAGTG | TGGATGCTCTCATCAGGACAG |
| TNF-α | CAGGCGGTGCCTATGTCTC | CGATCACCCCGAAGTTCAGTAG |

Table S2 LE and EE of BSA loaded SAHA hydrogel microspheres prepared at different formulations

| No. | BSA (mg/mL) | SA (w/v%) | CaCl_2_ (w/v%) | SA: CaCl_2_(V: V) | EE (%) | LE (%) |
| --- | --- | --- | --- | --- | --- | --- |
| 1 | 2 | 1 | 2 | 3：1 | 51.70 | 8.46 |
| 2 | 2 | 1 | 5 | 3：1 | 67.35 | 8.38 |
| 3 | 2 | 1 | 10 | 3：1 | 82.65 | 5.74 |
| 4 | 2 | 1.5 | 2 | 3：1 | 66.35 | 7.89 |
| 5 | 2 | 1.5 | 5 | 3：1 | 69.46 | 6.57 |
| 6 | 2 | 1.5 | 10 | 3：1 | 63.50 | 4.33 |
| 7 | 2 | 2 | 2 | 3：1 | 75.68 | 6.78 |
| 8 | 2 | 2 | 5 | 3：1 | 88.97 | 6.50 |
| 9 | 2 | 2 | 10 | 3：1 | 75.60 | 4.46 |
| 10 | 2 | 2 | 2 | 2：1 | 56.14 | 4.49 |
| 11 | 2 | 2 | 2 | 1：1 | 59.16 | 5.73 |
| 12 | 1 | 2 | 2 | 3：1 | 79.41 | 3.53 |
| 13 | 3 | 2 | 2 | 3：1 | 56.69 | 8.82 |


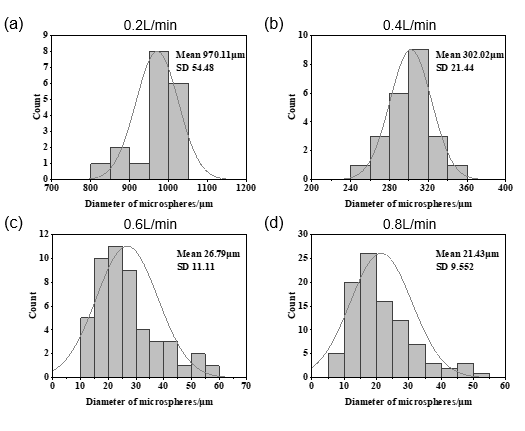


Figure. S1 Histograms of particle size distribution for different gas flow rates.


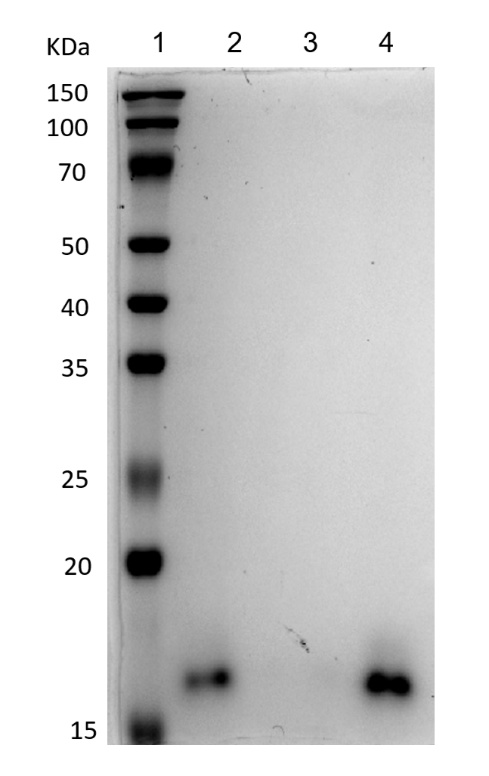


Figure. S2 SDS-PAGE pattern of molecular marker (lane 1), the released His-MANF with the protection of MSH@E after exposed SGF for 2 h and SCF for 6 h (lane 2,) His-MANF protein in SGF (lane 3) and the purified His-MANF protein (lane 4).


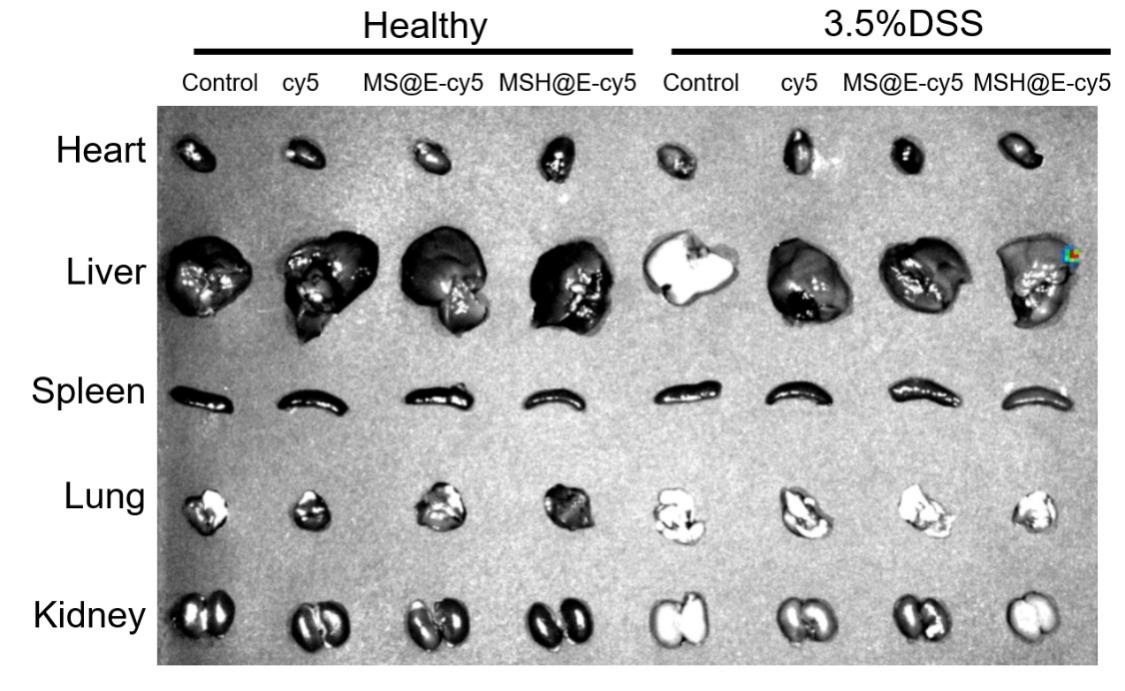


Figure. S3 Fluorescence imaging of heart, liver, spleen, lung, kidney of healthy mice and colitis mice with different treatment


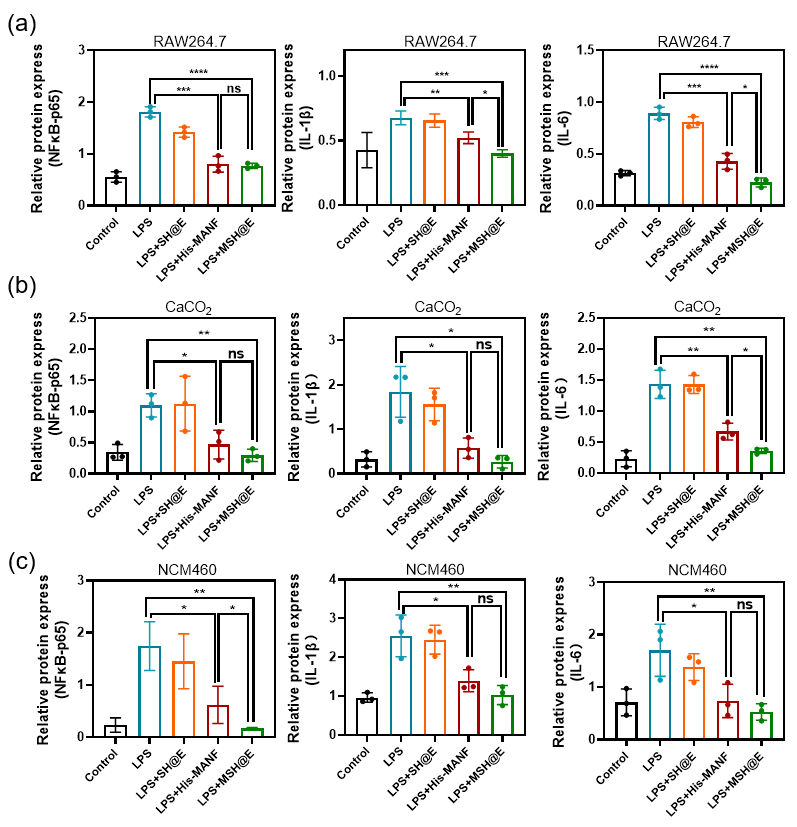


Figure. S4 The normalized gray value of NF-κB p65, IL-6 and IL-1β in WB quantified by using Image J software. (ns: not significant, ^*^*p* < 0.05, ^**^*p* < 0.01, ^***^*p* < 0.001, ^****^*p* < 0.0001)


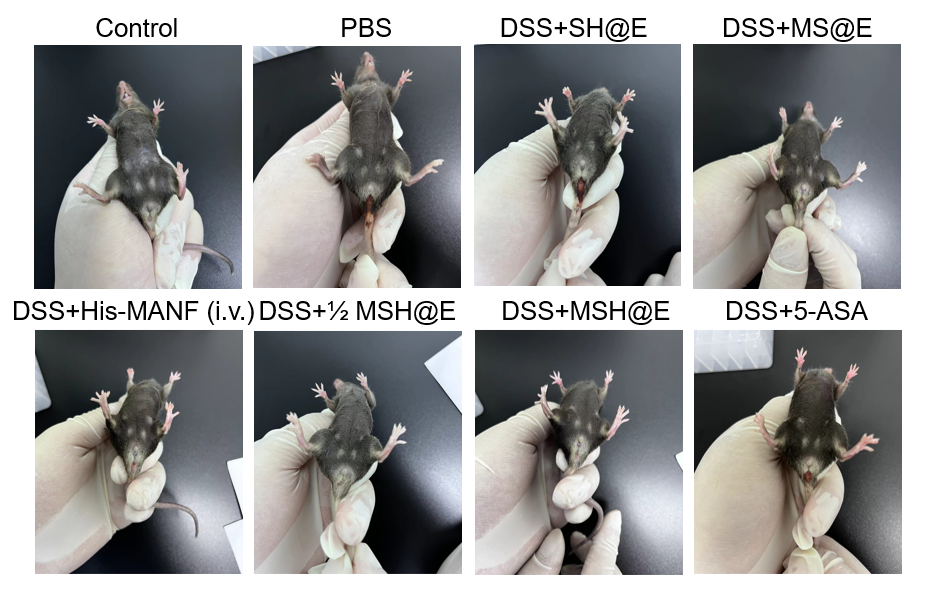


Figure. S5 Representative photographs of mice anus in different groups


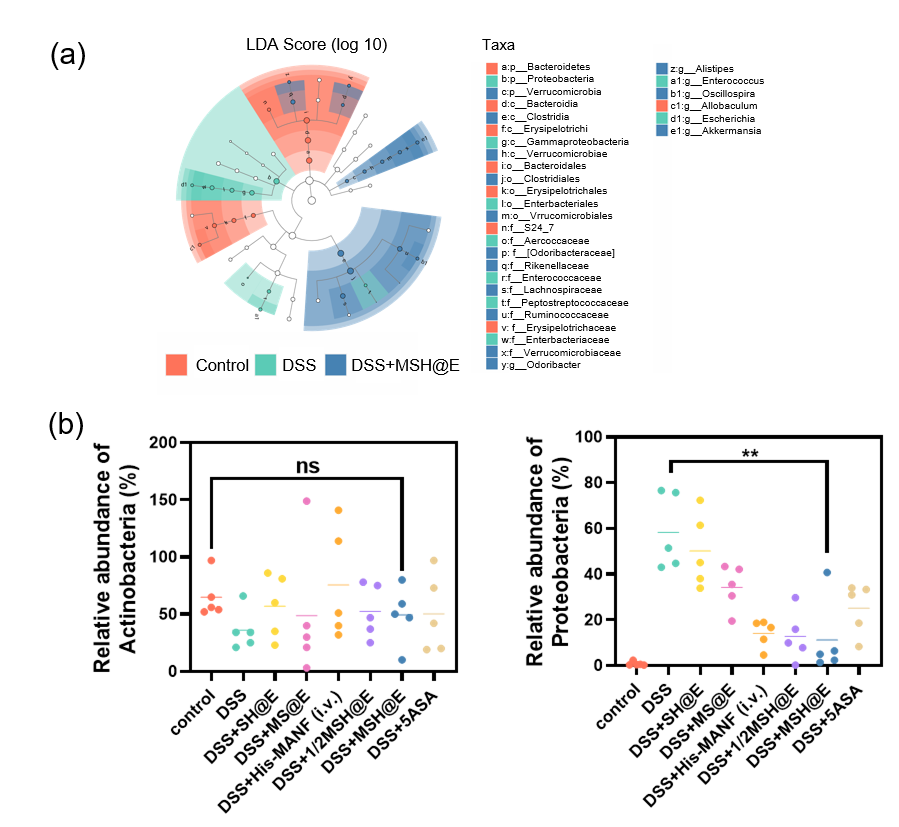


Figure. S6 (a) Significant differences in the abundance of gut microbiota between groups. The brightness of each point is proportional to its effect. (b) Relative abundance of Actinobacteria and Proteobacteria (n= 5, ns: not significant, ***p* < 0.01)
